# Searching for Survivors through Random Human-Body Movement Outdoors by Continuous-Wave Radar Array

**DOI:** 10.1371/journal.pone.0152201

**Published:** 2016-04-13

**Authors:** Chuantao Li, Fuming Chen, Fugui Qi, Miao Liu, Zhao Li, Fulai Liang, Xijing Jing, Guohua Lu, Jianqi Wang

**Affiliations:** 1 Department of Biomedical Engineering, Fourth Military Medical University, Xi’an, China; 2 Shaanxi University of Technology, Hanzhong, China; National Research Council, ITALY

## Abstract

It is a major challenge to search for survivors after chemical or nuclear leakage or explosions. At present, biological radar can be used to achieve this goal by detecting the survivor’s respiration signal. However, owing to the random posture of an injured person at a rescue site, the radar wave may directly irradiate the person’s head or feet, in which it is difficult to detect the respiration signal. This paper describes a multichannel-based antenna array technology, which forms an omnidirectional detection system via 24-GHz Doppler biological radar, to address the random positioning relative to the antenna of an object to be detected. Furthermore, since the survivors often have random body movement such as struggling and twitching, the slight movements of the body caused by breathing are obscured by these movements. Therefore, a method is proposed to identify random human-body movement by utilizing multichannel information to calculate the background variance of the environment in combination with a constant-false-alarm-rate detector. The conducted outdoor experiments indicate that the system can realize the omnidirectional detection of random human-body movement and distinguish body movement from environmental interference such as movement of leaves and grass. The methods proposed in this paper will be a promising way to search for survivors outdoors.

## Introduction

Rescuing survivors is very difficult because it is not easy for rescuers to arrive at the accident site immediately after an explosion at a chemical factory or a disaster at a nuclear power plant. If the survivors can be found quickly, their rescue and the efficiency at which the wounded are treated will be greatly improved. Although there are techniques that use cameras to monitor physiological signals, e.g., a high-definition camera and Microsoft Kinect sensor with advanced algorithms can detect respiration [1, 2], they have inherent limitations [3]. Methods using a camera are susceptible to the sight distance, e.g., at night or in places with smoke or fog, and their detection ability is limited. Furthermore, a thermal infrared camera can detect an object’s temperature, but there is a significant delay in determining whether the detected object has vital signs through the body temperature. Thus, it is difficult to reach the detection demand by only using camera detection technology to search for survivors. In order to meet this need, 24-GHz continuous wave (CW) radar is applied to detect humans outdoors.

A Doppler-radar motion-sensing system typically transmits a CW signal, which is reflected off a target and then demodulated in the receiver. It can detect the movement of an object by measuring the change in the phase between the object and the radar. On the basis of the Doppler principle, biological radar can detect body movement at a remote distance to identify vital signs [4]. Besides monitoring physiological signals in a noncontact and noninvasive manner, biological radar is also capable of penetrating a nonmetallic substance with a certain thickness. Thus, it has a wide variety of applications such as searching for human subjects under earthquake rubble or behind a barrier [5, 6]. An intelligent vehicle can be used to carry the radar to locate the survivors in a dangerous environment, thus improving the rescue efficiency.

At present, biological radar is often used to search for survivors by detecting their respiration signal [7, 8], and it has a stronger periodicity in comparison with random movement. However, there are disadvantages associated with using the respiration signal as the sole criterion to determine whether the detected object has vital signs. Because of the rescue urgency, only several minutes are spared during the search; if the object has frequent random body movements during detection, the detected respiration signal is irregular, which may be easily determined to be nobody at the site. In particular, the random body movements may increase when the injured survivor has unstable vital signs, thus misjudgment by the life recognition system will increase as well. The authors in [7] do not consider the body movement of an object under the premise of determining vital signs by assuming that the detected object is in a comatose state. Previous studies have mainly considered methods for eliminating the influence of body movement on the measurement accuracy of the respiration frequency. For example, [9] presents a different method using two radar sensors, [10] introduces an injection locking radar array to eliminate random body movement, [3] acquires a body movement signal via a mobile phone to realize phase compensation to eliminate random body movement, [11] presents a nonstationary approach to vital-sign detection and characterization in the presence of noise and body movement interference, and [12] applies a radar to detect the body movement and respiration of the operator to eliminate the influence of the body movement on the measurement of the respiration frequency. However, it is often difficult to know where the survivors are when searching for them outdoors. There is no condition to use several radars to detect the same object or a camera to detect the body movement; besides, the working capacity of the camera greatly decreases at night. A single threshold is used to determine whether the detected object has a random body movement signal, which may cause a high rate of misjudgment owing to the influence from the movement of leaves and grass. Thus, it is difficult for single-antenna radar to detect the respiration of a human body when random body movement exists. At present, there are few studies involving the detection of humans to identify vital signs by detecting random body movement since it is difficult to distinguish it from the movement of leaves and grass. Since the body movement signal has no periodicity, it is difficult to distinguish the body movement signal from the instantaneous interference of radar, where the existing common frequency demodulation methods and Fourier analysis would not work in the presence of high noise and environmental motion interference.

By considering the random position of an object relative to the radar and the shortcomings of single-antenna radar, an omnidirectional detection system consisting of four 24-GHz CW radar antennas is proposed in this paper to search for survivors. The radar used here is an IPM-165 radar (InnoSenT, Germany), which features a small size (20 × 20 × 6 mm^3^), small power consumption (0.2 W), and long coverage range (15 m). Disturbances caused by the movement of leaves and grass can be detected by the radar array, and human respiration or other body movement covered can also be detected. Body movement can be distinguished from environmental disturbances through a comparison of the differences among different radar echoes. The identification of the human-body-movement signal provides a new standard for determining whether the detected object has vital signs. When a person’s head or feet are in the front of the main lobe of the radar, which blocks the respiration part, the respiration signal in the radar echo becomes weak; however, the radar can still detect a random body movement to determine whether the object has vital signs. Such a method greatly improves the judgment accuracy of whether the detected object has vital signs.

There are many studies on improving the detection accuracy by a radar array or an antenna array. For example, [13] applies four radars to an experimental object in four directions to eliminate the influence of random body movement on respiration and heartbeat measurement in combination with a certain algorithm. [12] applies two radars (one for the object and the other for the operator) to eliminate the influence of the operator’s body movement on the measurement of the respiration signal of the object. [2] applies two emission frequencies for the radar antenna to eliminate the null points of CW radar and improve the detection accuracy of the respiration signal. In all of these studies, the position of the object relative to the radar is fixed, and the radar can realize good detection results through the adjustment of the angle. In this study, the position of the object relative to the radar is random; that is, the operators do not know the specific position of the object in the radar array and cannot realize good detection by adjusting the radar direction. Thus, the research is difficult.

This paper is organized as follows. The biological radar array for omnidirectional vital-sign monitoring is presented in Section 2. The four-channel identification technology for random body movement is introduced in Section 3. The experimental results are presented and analyzed to validate the effectiveness of the omnidirectional radar system and the method for body movement identification in Section 4. It is shown that the 24-GHz CW radar system can realize omnidirectional searching, and the identification technology for random body movement can distinguish random human-body movement from the movement of leaves and grass.

## Material and Methods

### 2.1 Omnidirectional biological radar system

**Fig 1:**
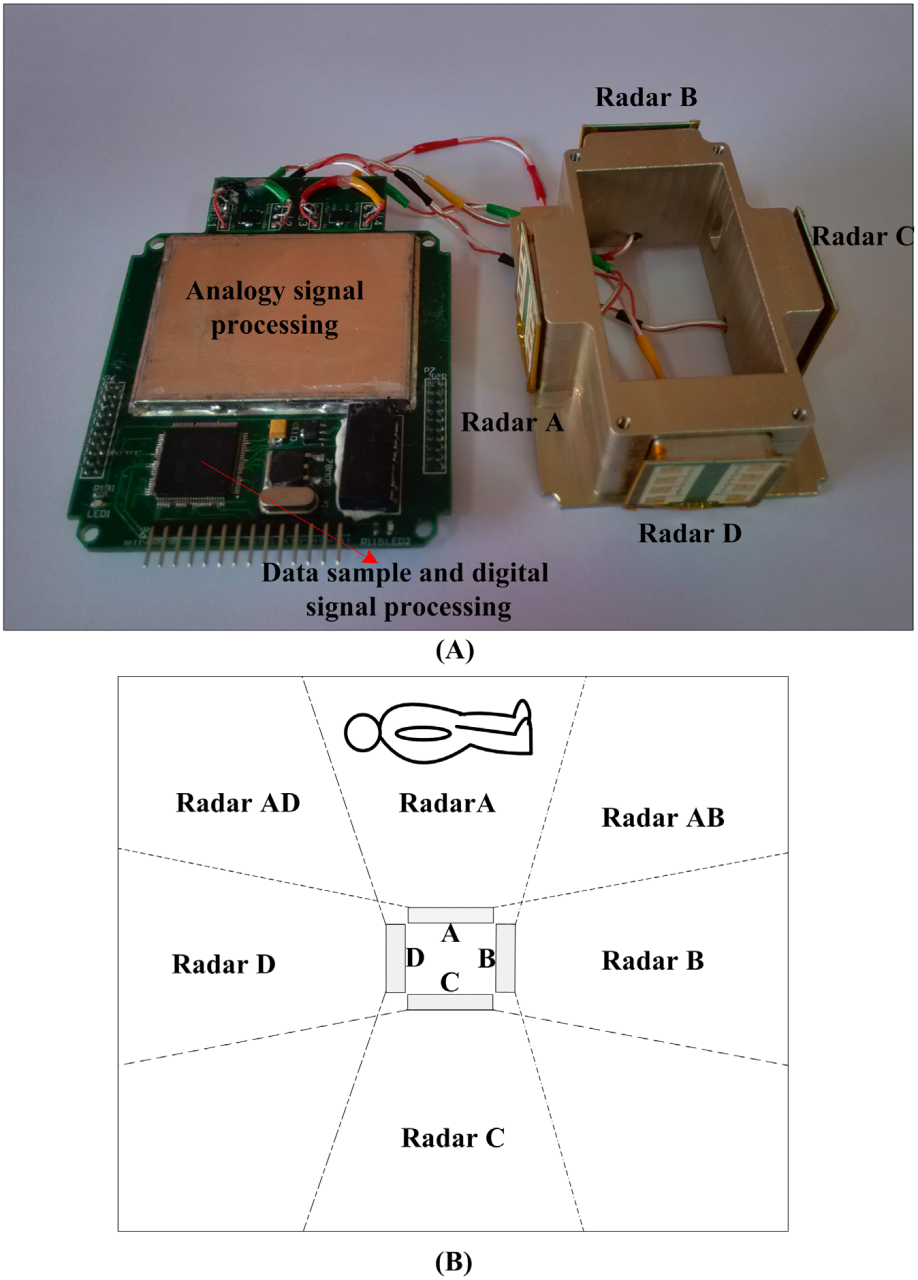
Omnidirectional life detection radar system. (A) Photograph of the omnidirectional life detection radar system. (B) Schematic of the radar coverage.

In a CW radar system, a sine wave is transmitted to a target, where it is reflected, and the back-reflected wave reaches the receiving antenna with a time difference that depends on the distance to the target, i.e., the phase difference between the transmitted and received signals is directly proportional to the motion of the target. Then, the movement information of the target can be extracted by processing the radar echo signal. When biological radar is used to search for survivors, the position of the detected object is unknown, the object may be at any position from the radar system, and biological radar probably cannot detect the object. In order to detect the object, intermittent rotation and detection of a single radar can be used; however, it has shortcomings such as a complex mechanical structure and low detection speed. In this study, an omnidirectional radar array consisting of four channels and four radars is used to realize omnidirectional searching, which effectively overcomes the shortcomings of a single radar, such as the intermittent rotation, complex mechanical structure, low detection speed, and large power consumption. The omnidirectional life detection radar system is shown in Fig 1.

Fig 1 shows a photograph of the omnidirectional life detection radar system and a schematic of the radar coverage. The study was approved by the Ethical Committee of the Fourth Military Medical University, and all participants provided their written informed consent before the experiment. As shown in Fig 1A, the left refers to the four-channel radar analog-signal preprocessing circuit and digital-signal acquisition and processing circuit. The band-pass range of the filter designed in this study is 0.05–4 Hz, the total amplification gain of multiamplifier is 60 dB, and the analog-signal preprocessing circuit of different radars is independent. The digital circuit adopts an STM32F407 chip for programming to carry out data sampling and digital-signal processing. In Fig 1A, the right refers to four radars, which are labeled Radar A, Radar B, Radar C, and Radar D. The effective body movement detection distance of the sensor is 10 m. The −3-dB horizontal coverage of a single radar is 80°, the −5-dB horizontal coverage is 90°, and the −3-dB vertical coverage is 30°. The four radars face different directions. The coverage of each radar is shown in Fig 1B. The human body is within the coverage of Radar A and can be within the common coverage of two radars at most. When searching for survivors, the four radars operate simultaneously. Since the center frequencies of the radars are slightly different, there is no same-frequency interference when they operate simultaneously.

Before using the radar to detect survivors, the safety issues regarding human health should be taken into account. It is generally concurred that long-term exposure to electromagnetic waves can be delicate or even dangerous [14]. During the search for survivors, each detected object is exposed to radiation from the radar for a short time. However, in research, the experimental volunteers are exposed to radiation from the radar for a long time. For example, the human experiments are conducted for 3 min each time in this study; thus, the safety of biological radar must be taken into consideration. According to the calculation of the power of the electromagnetic radiation emitted from biological radar in [2], the maximum acceptable density is approximately 12.67 mW/m^2^ when the distance between the 24-GHz biological radar sensor and the human body is 20 cm. The maximum allowable power density level is 10 W/m^2^ [15] for human exposure at frequencies from 10 to 300 GHz. The maximum acceptable power density is much lower than the maximum allowable accepted safe power density level. Therefore, the radar sensor poses no risk to human health.

### 2.2 Random Body Movement Detection

The movement of the chest due to breathing can be considered as a type of micromotion. Any random body movement during recording may cause considerable distortion in the recorded signal indoors. In an outdoor environment, the movement of leaves and grass may also cause considerable distortion in the recorded signal. Moreover, these two types of distortion have no differences in the radar echo signal in one radar channel. It is difficult to distinguish random body movement from the movement of leaves and grass by either the energy or frequency spectrum. Human respiration movement often ranges from 4 to 12 mm, whereas random body movement is obviously greater than respiration movement; thus, instantaneous sudden changes in the intensity and frequency of radar echo signal may occur when the object has random body movement. When a survivor is injured, the body movement signal caused by twitching, waving, or shouting is often referred to as an intermittent signal, and the change in the short-term variance of the radar echo signal can be used to identify random body movement. If the entire detected environment is known, the accuracy of radar detection can be improved. A radar system consisting of several radars solves the problem of a random object position and also provides new method for eliminating random noise in the environment.

Firstly, we define the four radars as Radar A, Radar B, Radar C, and Radar D in a clockwise direction. *x*_*A*_(*i*) indicates the signal of Radar A, *x*_*B*_(*i*) indicates the signal of Radar B, and so on. The sampling frequency of system is 20 Hz. Since the random body movement of the detected object is often intermittent, it is necessary to use a sliding variance to identify the change caused by body movement. The calculation of the sliding variance is as follows:

$$\delta_{A}(n)=\sqrt{\frac{\sum_{i=n-L+1}^{n+L/2}x_{A}{\left(i\right)}^{2}}{L+L/2}-(\frac{\sum_{i=n-L+1}^{n+L/2}x_{A}(i)}{L+L/2}}{)}^{2},n=L,2L,3L,4L\ldots$$ (1)

where *δ*_*A*_(*n*) is the smooth average deviation of *x*_*A*_(*i*), and it is computed using a window of length 40 (approximately 2 s). In order to improve the calculation speed and reduce the memory usage of the processor chip, step *L* of *n* is set as 40.

**Fig 2:**
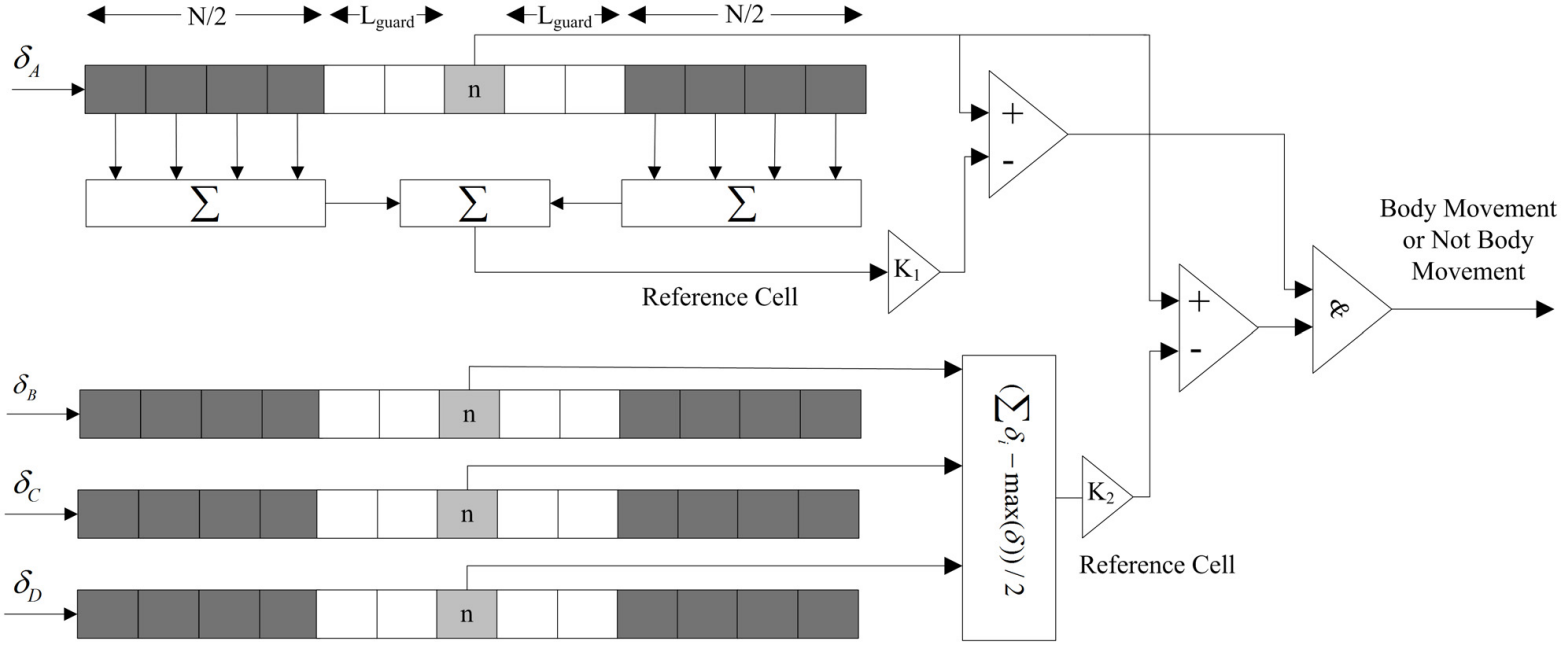
CFAR processor for body movement detection.

The background noise of the radar echo in different environments is not the same. The cell-averaging constant false alarm rate (CA-CFAR) method is used to maintain the stability of the identification of vital signs [16]. In this study, a two-dimensional CA-CFAR method is proposed to identify random body movement outdoors, similar to the method for identifying apnea [17], in combination with the structural characteristics of four radars. This procedure is illustrated in Fig 2.

When there is a body movement message contained in the radar echo signal, the instantaneous variance is large, and it can be preliminarily determined through a comparison of the variance whether body movement exists in the radar echo at a certain time. As shown in Fig 2, the average value of *N* points before and after the measurement point is used as the variance of the background noise. The background noise variance *Z*_1*A*_(*n*) of Radar A at the *n*th point is calculated by

$$Z_{1A}(n)=\frac{1}{N}(\sum_{i=n-N/2-Lguard}^{n-Lguard}\delta_{A}(i)+\sum_{i=n+N/2-Lguard}^{n+Lguard+N/2}\delta_{A}(i))$$ (2)

where *N* is the size of the window used in the averaging, and *L*_*guard*_ is the number of guard cells around the reference *n*th sample left to prevent edge effects. In this work, *N* is set to 4, and *L*_*guard*_ is set to 4.

By comparing the variance of the identification point and the background variance near the identification point, it can be preliminarily determined whether there is random body movement or an environmental disturbance at the identification point. It is necessary to distinguish the movement of leaves and grass from random body movement. As shown in Fig 1B, the target can be within the coverage of two radars at most; thus, the mean variance of the other two radars can be used as the background noise of the environment, which is calculated as follows:

$$Z_{2A}(n)=((\delta_{B}(n)+\delta_{C}(n)+\delta_{D}(n))-\text{max}(\delta_{B},\delta_{C},\delta_{D}))/2$$ (3)

where Z_2*A*_(*n*) indicates the background variance of the environment, where all radars are at 2*n* second; *δ*_*B*_(*n*), *δ*_*C*_(*n*), and *δ*_*D*_(*n*) are the variances of Radar B, Radar C, and Radar D, respectively, at the *n*th point; and channels A, B, C, and D have no particularity—when one channel is identified, the other three will be used as reference signals. Here, the maximum value among the three variances is removed during the calculation of the background noise because the object may exist within the common coverage of the two radars. For example, when the object exists in the coverage of Radar A, it may be covered by an adjacent radar. If Radar A acquires a human-body movement signal, its adjacent radar may also acquire the human-body movement signal; thus, the radar echo with two small variances in Eq (3) is used as the entire environmental background.

After the calculation of the one-channel background at the *n*th point and the environmental background at the *n*th point of the signal channel, it can be determined whether the detected object has random body movement by setting two thresholds to ensure that it has vital signs. For example, the calculation for identifying whether Radar A has random body movement at the *n*th point is as follows:

$$Index_{A}(n)=\left\{ \begin{array}{l} 1,\;(\delta_{A}(n)/Z_{1A}(n)>K_{1})\cap (\delta_{A}(n)/Z_{2A}(n)>K_{2})\\ 0,\;else \end{array}\right.$$ (4)

where *Index*_*A*_(*n*) = 1 indicates that Radar A has random body movement at the *n*th point, namely, at the 2*n* second; and *Index*_*A*_(*n*) = 0 indicates that Radar A has no random body movement at the *n*th point, namely, at the 2*n* second. *K*_1_ is the environmental background threshold of a single channel, and *K*_2_ is the entire environmental background threshold at the moment of identification. When the variance at the identification point conforms to the conditions of the two thresholds, it is determined that a person exists at the detected point. Through a large number of experiments, it is concluded that the system can realize a high identification accuracy when *K*_1_ = 1.45 and *K*_2_ = 1.65.

## Results and Discussion

**Fig 3:**
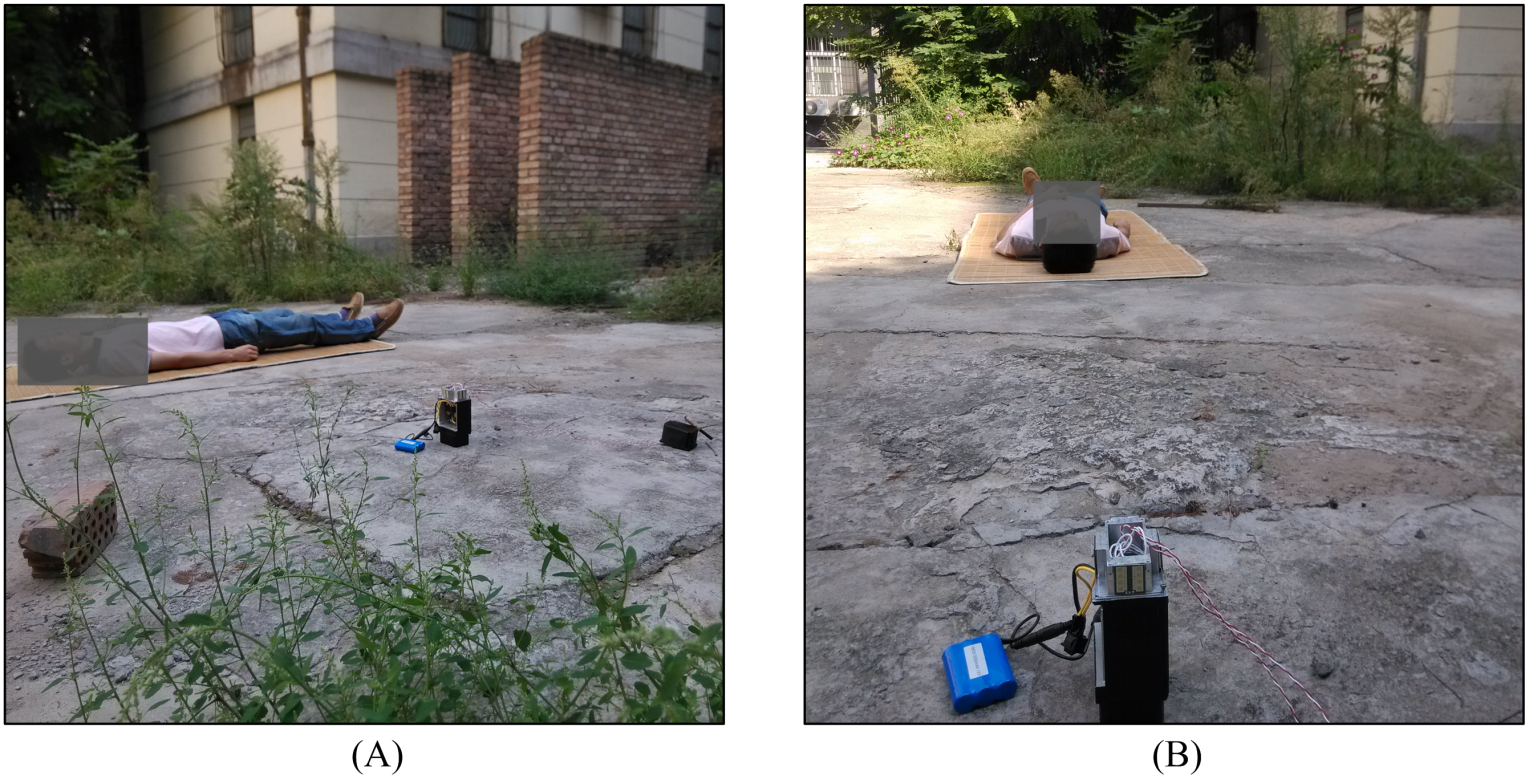
Photographs of the experimental scenes. (A) The object was within one radar’s coverage range, and the main lobe was directly facing the object's abdomen. (B) The object was within one radar’s coverage range, and the main lobe was directly facing the object's head.

In order to investigate typical situations through long-term monitoring, we performed measurements on three male volunteers (one 26-year-old, one 28-year-old, and one 30-year-old) who participated in the experiments for several positions and cases. The system sampling frequency is 20 Hz, and a 433-MHz CC1100 wireless communication module is used to send the acquired data and identification results to the computer. Each experiment is conducted for 3 min; every minute, the experimental object is told to make some body movements for approximately 3–6 s. In each experiment, the distance between the target and the radar array is approximately 2 m. In this study, the longest detection distance of the radar is approximately 10 m; to avoid disturbances, data are recorded behind a wall that is located 30 m away from the object. The study focused on discriminating body movements from those of leaves and grass; thus, all experiments were carried out in windy and vegetated environments. According to the position of the human body relative to the radar, the experiments were mainly divided into three scenes. The first scene is that the main lobe of a radar covers the abdomen of the human body, as shown in Fig 3A. The second scene is that the detected object is between two radars. The third scene is that the feet or head are in front of the radar; thus, the abdomen movement caused by respiration is blocked by the head, shoulders, feet, or legs, as shown in Fig 3B. In the third scene, it is difficult to determine whether the detected object has vital signs through human respiration signal recognition since the respiration signal in the radar echo is weak and cannot be acquired.

**Fig 4:**
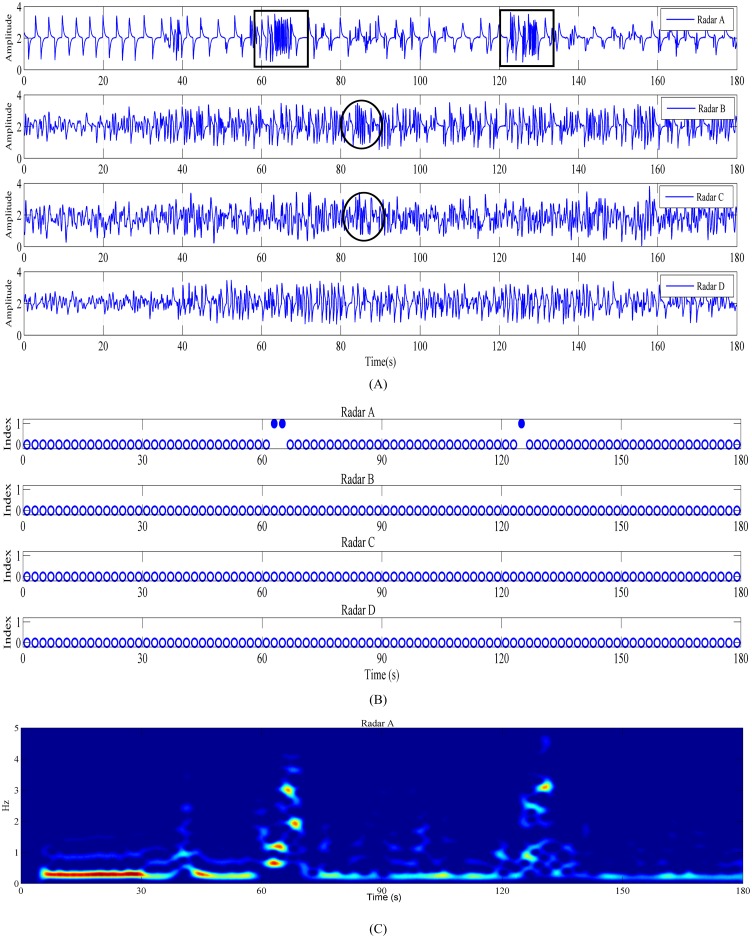
Detection results when a human body was within the coverage range of radar A, and the main lobe of radar A’s antenna was directly facing the human body’s abdomen. (A) Signals acquired by the four radars. (B) Body movement identification results. (C) Periodogram obtained from Radar A. The data can be acquired from the supporting information files.

The first experimental results are shown in Fig 4, and Fig 4A shows the time-domain waveforms of the four radars. The data can be acquired from the supporting information files. Radar A is blocked by the human body, the echo noise is small, and Radars B, C, and D have strong echo noise when there is no blockage by a human body. In Radar A, a human-body respiration waveform can be clearly observed when the detected object has random body movement at approximately 60 and 120 s, after which the signal varies violently, as shown in Fig 4A, which are underlined by black boxes. In Fig 4A, a strong signal change was also observed at around 40 s; this was because the detected object was coughing at that moment. Since the duration of coughing was short, the value of this point after calculating the sliding variance was not particularly prominent and thus was not recognized during recognition. Such short random human-body micromovements can be recognized by reducing the threshold, but the system’s antidisturbance capability to environmental movements will be reduced. Fig 4B shows the identification results of the body movement signal; the system identifies body movement at 60 and 120 s in the echo of Radar A. In Fig 4A, there is no random human-body movement, although the circled waveforms in the detected signals of Radars B and C vary violently. Further, no misjudgment is observed in Fig 4B. Fig 4C shows the periodogram obtained from Radar A; the signal frequency varies at 60 and 120 s, which is caused by body movement.

**Fig 5:**
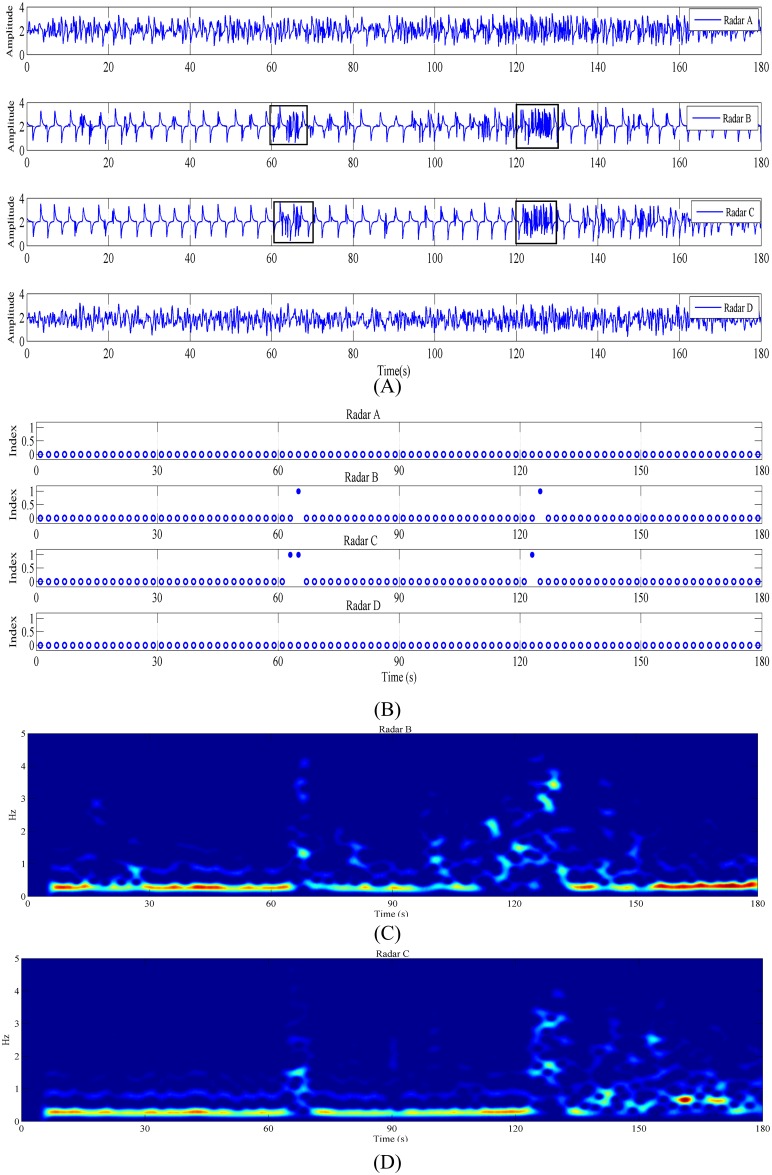
Detection results when a human body was within the common coverage range of two radars; the detected object was located between the radar-antenna side lobes of radar B and radar C. (A) Signals acquired by the four radars. (B) Identification results. (C) Periodogram obtained from Radar B. (D) Periodogram obtained from Radar C. The data can be acquired from the supporting information files.

The second experimental scene is similar to that shown in Fig 3A, but the detected object lies between two radar-antenna side lobes, that is, both of the radars can acquire the body movement of the detected object. The experimental results are shown in Fig 5, the data can be acquired from the supporting information files. The respiration waveform of a human body can be observed for Radars B and C in Fig 5A, and a violent variation in the radar signal caused by body movement can be observed at approximately 60 and 120 s, which is underlined by black boxes. Body movement is also identified by Radars B and C in Fig 5B. Fig 5C and 5D show periodograms obtained from Radars B and C, respectively. In the periodograms of Radars B and C, we can see the respiration frequency of the experimental object and the violent change in the echo frequency of the radar caused by random body movement at 60 and 120 s.

**Fig 6:**
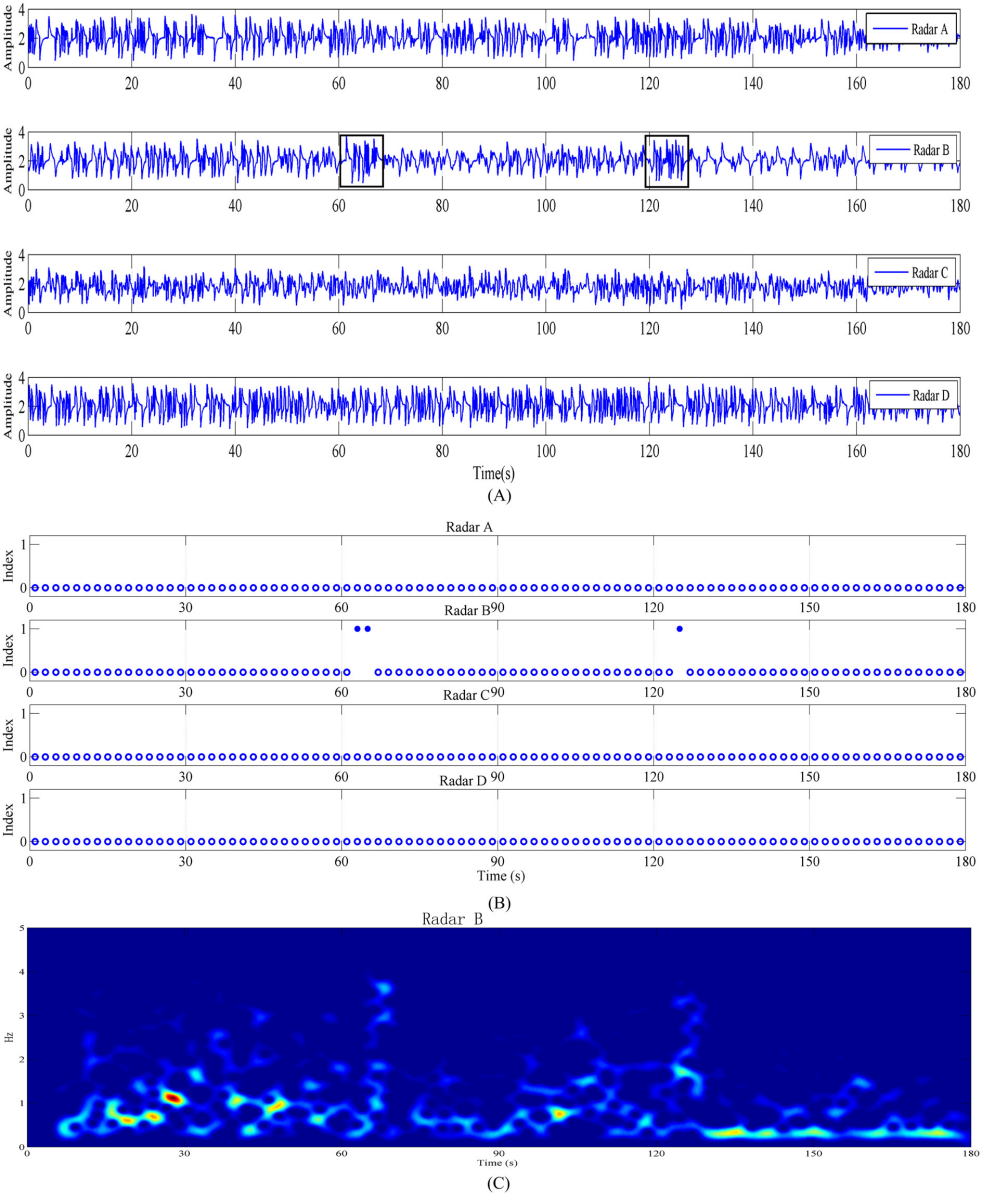
Detection results when a human body was within one radar’s coverage range and the main lobe of radar B’s antenna was directly facing the human body’s head. (A) Signals acquired by the four radars. (B) Body movement identification results. (C) Periodogram obtained from Radar B. The data can be acquired from the supporting information files.

The third experimental scene is shown in Fig 3B; the detected object lies flat, and the antenna of Radar B is in front of the head of the human body. The experimental results are shown in Fig 6, the data can be acquired from the supporting information files. Fig 6A shows the time-domain waveforms of the four radars. In this scene, the area of the human body blocking the radar is not greater than that in the first experimental scene; thus, a large amount of noise in the radar echo enters it. Owing to blocking by the head and shoulders, it is difficult to observe the respiration signal directly in the radar echo. Violent variations in the signal of Radar B caused by body movement can be observed at approximately 60 and 120 s, which are underlined by the black boxes in Fig 6. However, the method for identifying random body movement can identify the body movement signal in the echo of Radar B, as seen in the results in Fig 6B and the body movement signal in Fig 6A. Fig 6C shows the periodogram obtained from Radar B; there is a change in the frequency caused by body movement at 60 and 120 s, but this change is not obvious relative to the entire frequency distribution. However, the proposed method can still accurately identify random human-body movement.

## Conclusions

This paper presents an omnidirectional life detection system consisting of four 24-GHz CW biological radars for searching for survivors outdoors. The omnidirectional radar array can be used to effectively improve the searching efficiency and the identification accuracy of vital signs. The radar array provides a new means for the identification of vital signs, viz. identification of random body movement. In previous life detection studies, human respiration identification is always used to find survivors. However, when the radar system is used to detect humans, the method used to identify respiration is invalid if the object to be detected has random body movements. Random body movement is also an important indication that the human body has vital signs. In this study, the CA-CFAR method is used to identify random body movement successfully in an environment containing the movement of leaves and grass on the basis of the characteristic that multichannel radar can calculate the environmental background. In particular, when the radar is located in front of the human head or feet and the respiration of the object cannot be detected, it can be determined whether the object has vital signs by identifying the random body movement. Thus, the method for identifying random body movement presented in this paper can effectively improve the detection accuracy of vital signs via biological radar.

However, there will still be some issues when adopting the current algorithm. For example, the radar’s coverage range is related to environmental disturbances, where the radar’s effective coverage range will be reduced when the environmental disturbances increase. In addition, the method proposed in this study is only applicable for the detection and recognition of single objects; its accuracy will be reduced when there are multiple objects within in the radar’s coverage range. Aiming at the above problems, further investigations will be carried out in the future.

## Supporting Information

S1 Dataset Data underlying the study. (RAR)
